# The Evolutionary Maintenance of Amino Acid Prototrophy in *Escherichia coli*

**DOI:** 10.1093/gbe/evag209

**Published:** 2026-08-22

**Authors:** Otto Lindahl, Talía Berruga-Fernández, Jivanti Soekhoe, Douglas L Huseby, Diarmaid Hughes

**Affiliations:** Department of Medical Biochemistry and Microbiology, Biomedical Center, Uppsala University, Uppsala S-751 23, Sweden; Department of Medical Biochemistry and Microbiology, Biomedical Center, Uppsala University, Uppsala S-751 23, Sweden; Uppsala Antibiotic Center, Uppsala University, Uppsala, Sweden; Department of Medical Biochemistry and Microbiology, Biomedical Center, Uppsala University, Uppsala S-751 23, Sweden; Department of Medical Biochemistry and Microbiology, Biomedical Center, Uppsala University, Uppsala S-751 23, Sweden; Department of Medical Biochemistry and Microbiology, Biomedical Center, Uppsala University, Uppsala S-751 23, Sweden; Uppsala Antibiotic Center, Uppsala University, Uppsala, Sweden

**Keywords:** Muller's ratchet, auxotrophy, compensatory evolution, tandem amplification, amino acid biosynthesis, biosynthetic pathway diversion

## Abstract

*Escherichia coli* is a prototroph and can synthesize all twenty proteinogenic amino acids when required to grow in minimal medium. There are approximately sixty protein-coding genes individually essential for amino acid synthesis. This is a large mutational target for the accumulation of detrimental mutations. *E. coli* can rewire biosynthetic pathways in response to mutational damage, but the limits of this capacity are poorly understood. Here, to address evolutionary robustness, we asked whether and how the phenotypes of irreversible mutations causing auxotrophy could be suppressed or bypassed in the absence of horizontal gene transfer (HGT). Spontaneous suppressors could be selected for only ten of fifty-nine mutants tested (detection limit ∼7 × 10^−11^). Mechanisms of suppression included regional amplifications, mutations increasing gene or operon expression, mutations relaxing enzyme specificity, and mutations causing biochemical pathway diversions. Overall, the data show that spontaneous suppression of auxotrophy caused by an irreversible mutation is an evolutionary survival mechanism relevant only to a minority of the genes essential for amino acid synthesis. As a consequence, the essential genetic foundations for amino acid prototrophy are expected to be degraded over time by mutations (Muller's ratchet) and metabolic rewiring alone will be insufficient to counteract this effect. This implies that maintaining phenotypes, including prototrophy in *E. coli*, and potentially other bacterial species, is likely to be reliant on HGT of housekeeping genes to counteract the effects of inevitable mutational inactivation. Accordingly, chromosomal HGT in bacteria may be critical for survival across diverse environmental niches.

## Graphical Abstract

**Graphical Abstract:**
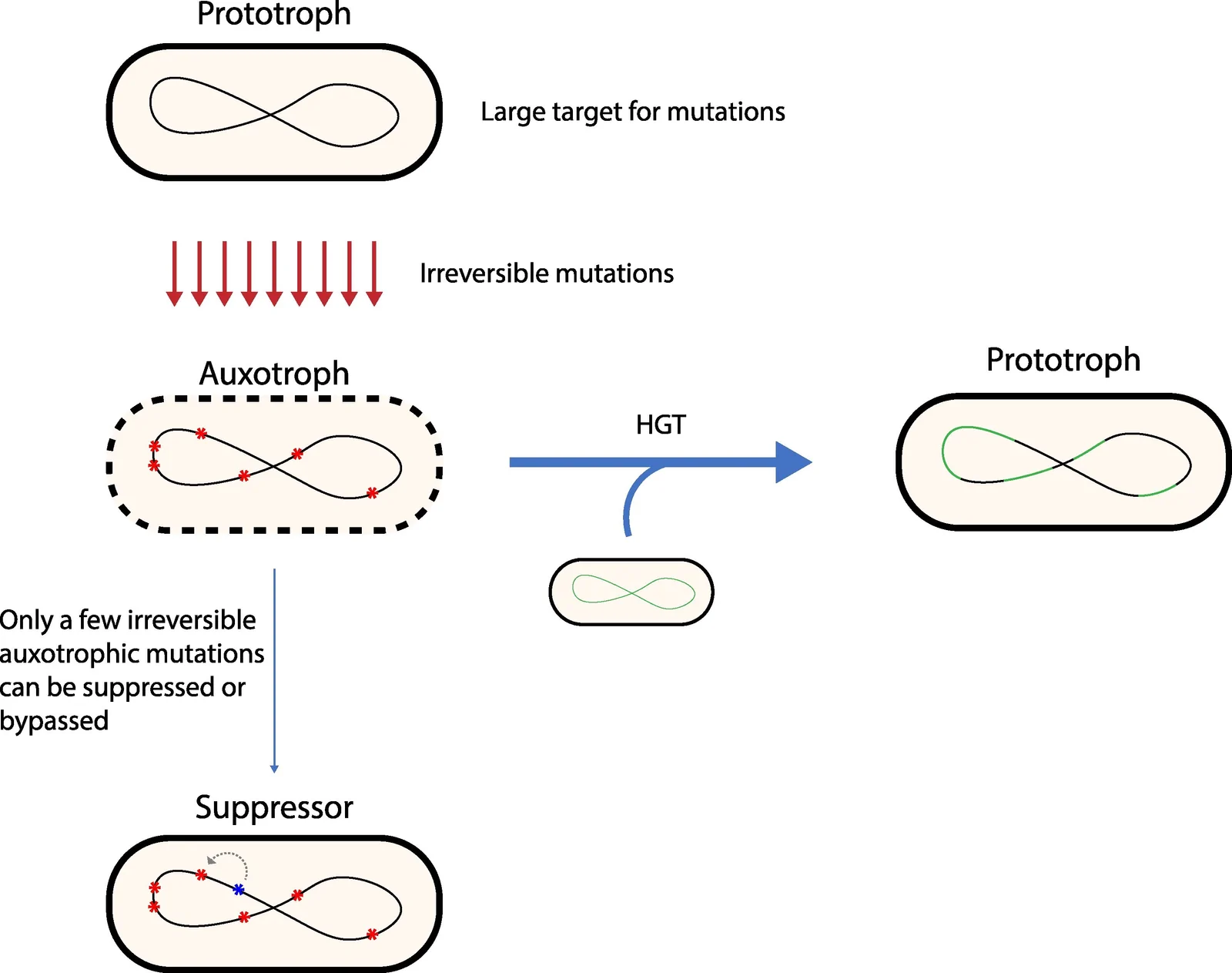

Significance How has *Escherichia coli*, in the face of continuous mutational pressure, maintained its genetic capacity to make all 20 amino acids required for protein synthesis and avoided becoming an auxotroph? We tested experimentally whether the effects of irreversible mutations inactivating each of 59 genes essential for amino acid biosynthesis could be reversed by compensatory mutations. Only in 10 of the 59 cases could compensatory mutations restore prototrophy (typically by mutations rewiring biosynthetic pathways or amplifying trace activities). We conclude that amino acid synthesis capacity (prototrophy) in *E. coli* is maintained over evolutionary timescales by horizontal gene transfers, restoring functions lost by accumulation of detrimental mutations.

## Introduction

*Escherichia coli* encodes the metabolic capability to support bacterial growth on a defined minimal medium of inorganic salts, such as M9, supplemented with a single carbon source such as glucose (Elbing and Brent 2019). The “Keio collection” of single-gene deletion mutants constructed in *E. coli* K-12 strain W3110 (Baba et al. 2006) is a useful tool for interrogating the essentiality and evolvability associated with any subset of those genes. A previous study identified 62 strains in the Keio collection unable to grow on M9-glucose because the knockout mutations affected protein-coding genes essential for amino acid metabolism (Patrick et al. 2007). They showed that those deletion mutants affecting biosynthetic genes could be suppressed by expression of the same gene from a multicopy plasmid (referred to as self-complementation or self-rescue (Patrick et al 2007), equivalent to acquiring the missing gene by horizontal gene transfer (HGT). They also showed that the auxotrophic phenotype of 11 of the mutants could be suppressed by the expression of single non-cognate genes from a multicopy plasmid (Kitagawa et al. 2005; Patrick et al. 2007). That study showed that in principle overexpression of non-cognate genes could provide an evolutionary backup mechanism to overcome the detrimental effects of mutations affecting some steps in amino acid metabolic pathways. However, the study left open whether, how frequently, and by which mechanisms, suppression of a genetically irreversible auxotrophy is possible by spontaneously occurring mutational events in the absence of horizontal genetic transfer (HGT).

As part of a study into the formation of bacterial chromosomal hybrids, we sought to identify amino acid auxotrophic mutations in *E. coli* that could not be suppressed except by horizontal genetic transfer (Berruga-Fernández et al. 2026). We observed that a constructed deletion of *proB* could be suppressed by spontaneous mutations in GlnA (glutamine synthetase), an enzyme that feeds the glutamine and proline pathways with a common intermediate (Medina-Carmona et al. 2023; Lindahl et al. 2024), and also by inactivating mutations in *argD* (Lindahl et al. 2024). In the previous study exploring the possibility for overexpressed non-cognate genes to complement auxotrophy, no multicopy suppressors of Δ*proB* were identified (Patrick et al. 2007). This suggested that the mechanisms of spontaneous suppression might be significantly different from those caused by single gene overexpression. This prompted us to reexamine the amino acid auxotrophic strains in the Keio collection (Baba et al. 2006) with regard to spontaneous suppression mechanisms. Here we report on the frequencies and mechanisms with which deletion mutants causing amino acid auxotrophy can be suppressed by spontaneously occurring mutations. The results may have general significance to the evolutionary maintenance of essential gene functions in *E. coli* and suggest that HGT plays a critical role in supporting the ability of the species to occupy a diverse range of environmental niches.

## Results

### Amino Acid Auxotrophs in the Keio Collection of Single Gene Deletion Mutants

We initially reevaluated amino acid metabolism mutant strains (Patrick et al. 2007) from the Keio collection (Baba et al. 2006), by assaying their growth after 120 h incubation at 37 °C on M9-glucose agar medium in the presence and absence of the relevant amino acid. Our aim was to establish selection conditions that would allow the spreading of ∼10^9^ CFU per M9-glucose agar plate without a lawn of growth that would hinder the isolation of suppressors. We found that three mutants on the original list were not, or only partially, auxotrophic (Δ*metL*, Δ*thrA*, and Δ*pheA*). We reconstructed these three deletion mutants in the original W3110 parental strain (Baba et al. 2006) and confirmed their genotypes by whole genome sequencing. Both *metL* and *thrA* encode isozymes that catalyze the same steps in converting L-aspartate to L-homoserine (Jin et al. 2024) and although neither deletion mutant alone was auxotrophic, we showed that a constructed double deletion (Δ*metL*, Δ*thrA*) makes the strain auxotrophic (Fig. S1a). The Δ*pheA* mutant showed density-dependent growth on minimal medium (Fig. S1b) and while technically auxotrophic, was unsuitable for the selection of suppressors of auxotrophy from large populations of cells. Accordingly, we progressed 59 single gene deletion mutants for the selection of spontaneous suppressors of amino acid auxotrophy (Table S1).

### Selecting Spontaneous Suppressors of Auxotrophy

A minimum of four independent cultures of each of the validated amino acid auxotrophic strains were spread on M9-glucose agar and incubated at 37 °C for 5 d (120 h) to allow the growth of prototrophic mutants (mutant detection limit ≤ 7 × 10^−11^). We selected suppressor mutants for 10 of the 59 auxotrophic deletion mutants, at frequencies that ranged from 7.0 × 10^−11^ up to 3.6 × 10^−8^. After selection, suppressors were purified and validated by restreaking for single colonies on M9-glucose agar to confirm their ability to grow, then stored at −80 °C. Genomic DNA was prepared, and whole genome sequencing was used to identify genetic alterations associated with spontaneous suppression of auxotrophy (Table 1). Below we present the data related to the suppressors of these ten different amino acid auxotrophic mutants in alphabetical order.

**Table 1:** Selection frequency and genetic identification of suppressor mutations

Deletion	Frequency	Genetic mechanism of suppression
Δ*cysB*	3.5 × 10^−10^	*cysPUWAM* operon (promoter mutations)
Δ*glyA*	1.0 × 10^−8^	*kbl-tdh* operon (promoter mutations)
Δ*hisH*	6.7 × 10^−10^	*his* operon region amplification (10- to 30-fold)
Δ*ilvA*	1.1 × 10^−8^	*cysB, cysE, cysK, metB, serA, thrC,* (mutations)
Δ*ilvE*	7.0 × 10^−11^	*avtA* region amplification (50-fold)
Δ*leuL*	2.0 × 10^−8^	*leuL-leuA* (intergenic mutations)
Δ*metR*	1.4 × 10^−9^	*metR-metE* (intergenic mutations)
Δ*proA*	3.6 × 10^−8^	*argD* (inactivation mutations) *asd* region amplification (16-fold) *asd* (promoter mutations)
Δ*proB*	3.3 × 10^−9^	*glnA* (amino acid substitutions)
Δ*serB*	8.5 × 10^−9^	*his* operon region amplification (30-fold) *hisL-hisG* (intergenic mutations) *truA* (inactivation mutation)

### Suppression of Δ*cysB*

**Fig. 1.**
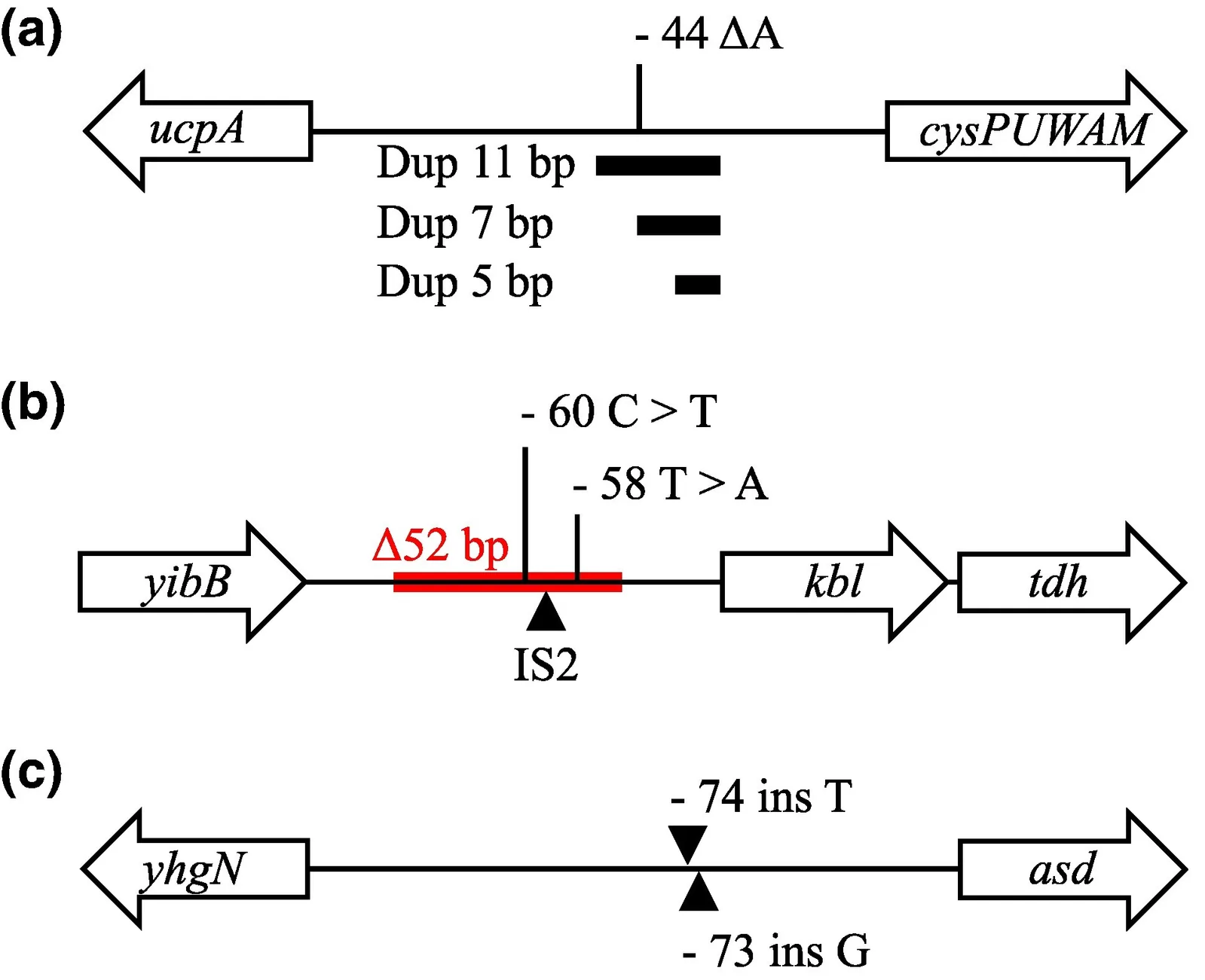
Mutations in promoter regions causing suppression of auxotrophy. a) Map of the region upstream of the *cysPUWAM* operon showing the locations of four mutations causing suppression of ΔcysB: three duplication mutations (thick black lines) and one single nt deletion, relative to the first nt of the start codon of *cysP*. b) Map of the region upstream of the *kbl-tdh* operon showing the locations of four mutations causing suppression of Δ*glyA*: one deletion mutation (thick line), one IS2 insertion, and two nt substitution mutations, relative to the first nt of the start codon of *kbl.* c) Map of the region upstream of *asd* showing the locations of two single nt addition mutations causing suppression of Δ*proA*, relative to the first nt of the start codon of *proA*.

CysB is a DNA-binding protein that controls sulfur assimilation in *E. coli* (van der Ploeg et al. 2001). It is the master transcriptional regulator for the cysteine regulon, controlling the uptake and assimilation of inorganic sulfate into L-cysteine, and is also required to activate transcription of the *cysPUWAM* operon for thiosulfate/sulfate uptake (Hryniewicz et al. 1990). The Δ*cysB* mutant is unusual among auxotrophs in that it grows slowly on minimal medium supplemented with cysteine, (Fig. S2), possibly reflecting the diversity of genes dependent on CysB protein for their expression (van der Ploeg et al. 2001). We selected four different suppressors on minimal medium (albeit still very slow growing) and, after whole genome sequencing, identified that each had acquired a mutation in the promoter region upstream of the *cysPUWAM* operon (Fig. 1a, and Table S2). Our hypothesis is that the suppressor mutations activate expression of the operon in the absence of CysB binding to the regulatory region, allowing bacteria to scavenge sulfate from the medium and support a small amount of growth without fully reversing the slow growth.

### Suppression of Δ*glyA*

Serine hydroxymethyltransferase (GlyA) converts L-serine to glycine (Stauffer 2004). We selected four independent suppressors of the Δ*glyA* mutant, all of which mapped in the promoter region upstream of the *kbl-tdh* operon (Fig. 1b). Each of the suppressor mutations (two different base pair substitutions, one 52 base pair deletion, one IS2 insertion) affects the −35 sequence of the promoter for the *kbl-tdh* operon (Fig. 1b, and Table S3). Both Kbl and Tdh are enzymes involved in reactions that result in the conversion of threonine to glycine (Stauffer 2004). Our hypothesis is that the selected suppressors work by increasing expression of the *kbl-tdh* operon. We cloned *tdh* onto pUC19 and confirmed that it was sufficient to suppress the Δ*glyA* auxotrophy (Fig. S3). It was shown previously that expression of *tdh* from a multicopy plasmid could suppress Δ*glyA* (Patrick et al. 2007).

### Suppression of Δ*hisH*

**Fig. 2.**
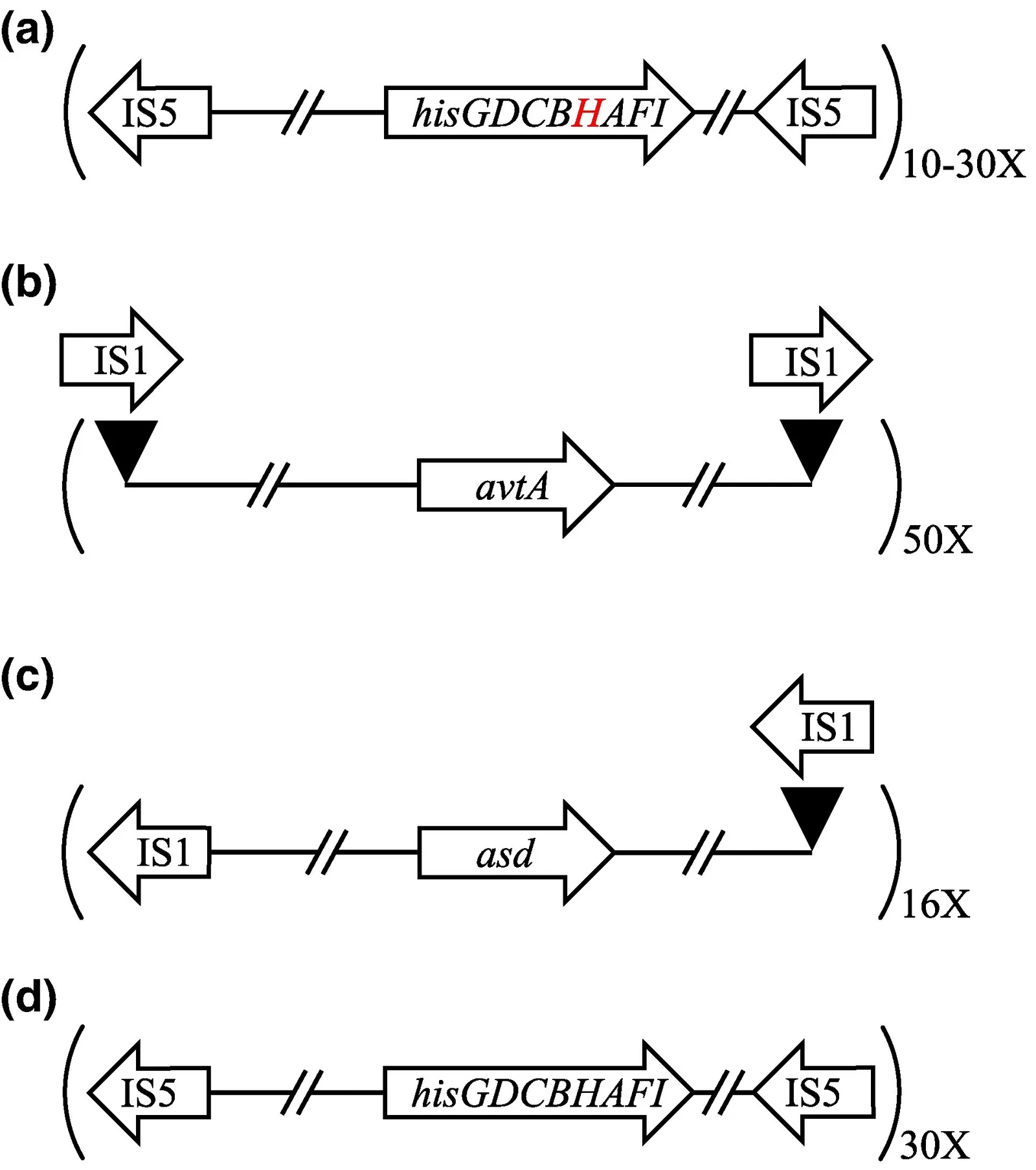
Amplification mutations causing suppression of auxotrophy. a) Suppression of Δ*hisH* caused by 10- to 30-fold amplification of a 35.7 kb region, including the histidine operon, by recombination between existing two chromosomal copies of IS5. b) Suppression of Δ*ilvE* caused by a 50-fold amplification of a 16 kb region, including *avtA* that occurred by recombination between two copies of IS1, each of which is newly inserted (indicated by inverted black triangles) to provide the substrate for homologous recombination and tandem amplification. c) Suppression of Δ*proA* caused by a 16-fold amplification of a 51 kb region, including *asd* that occurred by recombination between two copies of IS1, one of which preexisted, the other newly inserted (indicated by the inverted black triangle) to provide the substrate for homologous recombination and tandem amplification. d) Suppression of Δ*serB* caused by 30-fold amplification of the 35.7 kb region, including the histidine operon, between two preexisting copies of IS5 (same amplification as in section A.

The *hisH*-encoded enzyme (HisH) is one subunit of imidazole glycerol phosphate synthase, (HisFH, a 1:1 dimer) that converts L-glutamine to L-glutamate, catalyzing the fifth step in histidine biosynthesis. Isolated HisH has no enzymatic activity, but isolated HisF can catalyze the step using ammonia as a nitrogen donor in place of the normal physiological donor glutamine (Klem and Davisson 1993). We selected five independent suppressors (Table S4), four of which had a 10- to 30-fold amplification of a 35.7 kb chromosomal region that included the histidine operon (Fig. 2a). In each case the amplification was mediated by recombination between two copies of IS5. The remaining suppressor mutant had acquired a partial deletion in the Keio*-kan*-cassette that replaces *hisH* that may create a promoter sequence driving overexpression of the later genes in the operon, *hisAFI*. These results are in agreement with the observation that overexpression of *hisF* from a multicopy plasmid rescues the auxotrophy caused by deletion of *hisH* (Patrick et al. 2007). Accordingly, we suggest that the suppression depends on the increased production of HisF enzyme to drive the reaction normally carried out by the HisFH dimer enzyme (Fig. S4). We constructed a double mutant, Δ*hisH-*Δ*hisF* (Table S4), and found that spontaneous suppressors could no longer be selected (frequency < 7.4E−11), consistent with the hypothesis that suppression requires an active *hisF* gene.

### Suppression of Δ*ilvA*

IlvA is the threonine deaminase that carries out the first step in isoleucine synthesis, converting L-threonine to 2-oxobutanoate (Umbarger and Brown 1956). Fourteen independently selected suppressors of Δ*ilvA* were identified as carrying mutations in one of several different genes involved in amino acid metabolism: *cysE*, *cysK, cysB*, and *metB*, *serA*, *thrC* (Table S5). The three *cys*-genes in which suppressor mutations were isolated are functionally connected: the enzymes CysE (serine acetyltransferase) and CysK (O-acetylserine sulfhydrylase-A) form a multi-subunit cysteine synthase complex that synthesizes L-cysteine from L-serine (Benoni et al. 2017), while CysB is a transcriptional activator required for *cysK* expression (Kredich 2008). Accordingly, all three *cys-*genes in which suppressors were selected are directly involved in promoting the conversion of L-serine to L-cysteine. Mutation of *cysE* has been previously shown to reduce the intracellular concentration of cysteine, opening up a latent pathway that allows MetB (cystathionine γ-synthase) to convert O-succinyl-L-homoserine to 2-oxobutanoate which is then used to produce isoleucine (Cotton et al. 2020). Several of the suppressor mutations selected in *cysK* are predicted to reduce or abolish CysK enzymatic activity, (Table S5), suggesting that inactivating *cysK* might open up a mechanism to suppress the auxotrophy (Δ*cysK* is not itself auxotrophic). To test this, we constructed the double mutant Δ*ilvA* Δ*cysK* and found that it grew on minimal medium (Fig. S5c). Overall, these *cys-*gene suppressors are consistent with the hypothesis that reducing the conversion of L-serine to L-cysteine causes a metabolic pathway diversion, activating MetB to produce 2-oxobutanoate, resulting in bypass of the Δ*ilvA* auxotrophy (Cotton et al. 2020). We speculate that the suppressor mutation in *metB* may also increase the rate of MetB-dependent production of 2-oxobutanoate (Cotton et al. 2020). The mechanism of suppression of the mutation in *serA* (3-phosphoglycerate dehydrogenase) is less clear. One possibility is that because SerA provides the initial substrate (3-phosphoglycerate) for methionine and cysteine production (Stauffer 2004) that it indirectly activates the MetB-driven production of 2-oxobutanoate. Another possibility is that it alters the expression of a serine deaminase such as SdaA, potentially increasing its activity on threonine to produce 2-oxobutanoate (Cicchillo et al. 2004). A mechanism that could explain the suppressor mutation isolated in *thrC* is that mutations derepressing expression of the *thrCB* operon in *Bacillus subtilis* were shown to unmask a minor threonine dehydratase activity of ThrC, enabling the conversion of threonine to 2-oxobutanoate and suppressing Δ*ilvA* auxotrophy (Rosenberg et al. 2016). In addition to IlvA (threonine deaminase), there are two other enzymes capable of using threonine as a substrate in the production of 2-oxobutanoate leading to isoleucine: TdcB, catabolic threonine dehydratase (Umbarger and Brown 1957) and SdaA, L-serine deaminase 1 (Cicchillo et al. 2004). Overexpression of cloned *tdcB*, was previously shown to suppress Δ*ilvA* auxotrophy (Patrick et al. 2007). We addressed the possibility that some of the suppressors of Δ*ilvA* might depend on *tdcB* or *sdaA* to produce 2-oxobutanoate. To test this, we constructed strains carrying deletions of *tdcB* and *sdaA* together with Δ*ilvA* and an example of each of the different suppressor mutations. We assayed their continued ability to grow on minimal medium and found that all grew, showing that the suppressor phenotypes are independent of TdcB and SdaA activity (Fig. S5a to d).

### Suppression of Δ*ilvE*

IlvE is a transaminase B enzyme that carries out the final step in valine, leucine, and isoleucine biosynthesis (Salmon et al. 2006). Previous results (Patrick et al. 2007) showed that a multicopy plasmid carrying *avtA* could suppress the auxotrophy caused by either Δ*ilvD* or Δ*ilvE*. We selected a single spontaneous suppressor of Δ*ilvE*, in which a 50-fold amplification of a 16 kb chromosomal region that included *avtA* had occurred (Fig. 2b and Table S6). The amplification required the insertion of two IS1 elements into the chromosomal region that then provided the substrate for tandem amplification by homologous recombination. The requirement for two IS insertion events probably explains the rarity of this spontaneous suppression mechanism. The *ilv* operon itself is not amplified. We constructed an Δ*ilvE* strain carrying *avtA* cloned onto pUC19 and showed that it could grow on minimal medium (Fig. S6).

### Suppression of Δ*leuL*

**Fig. 3.**
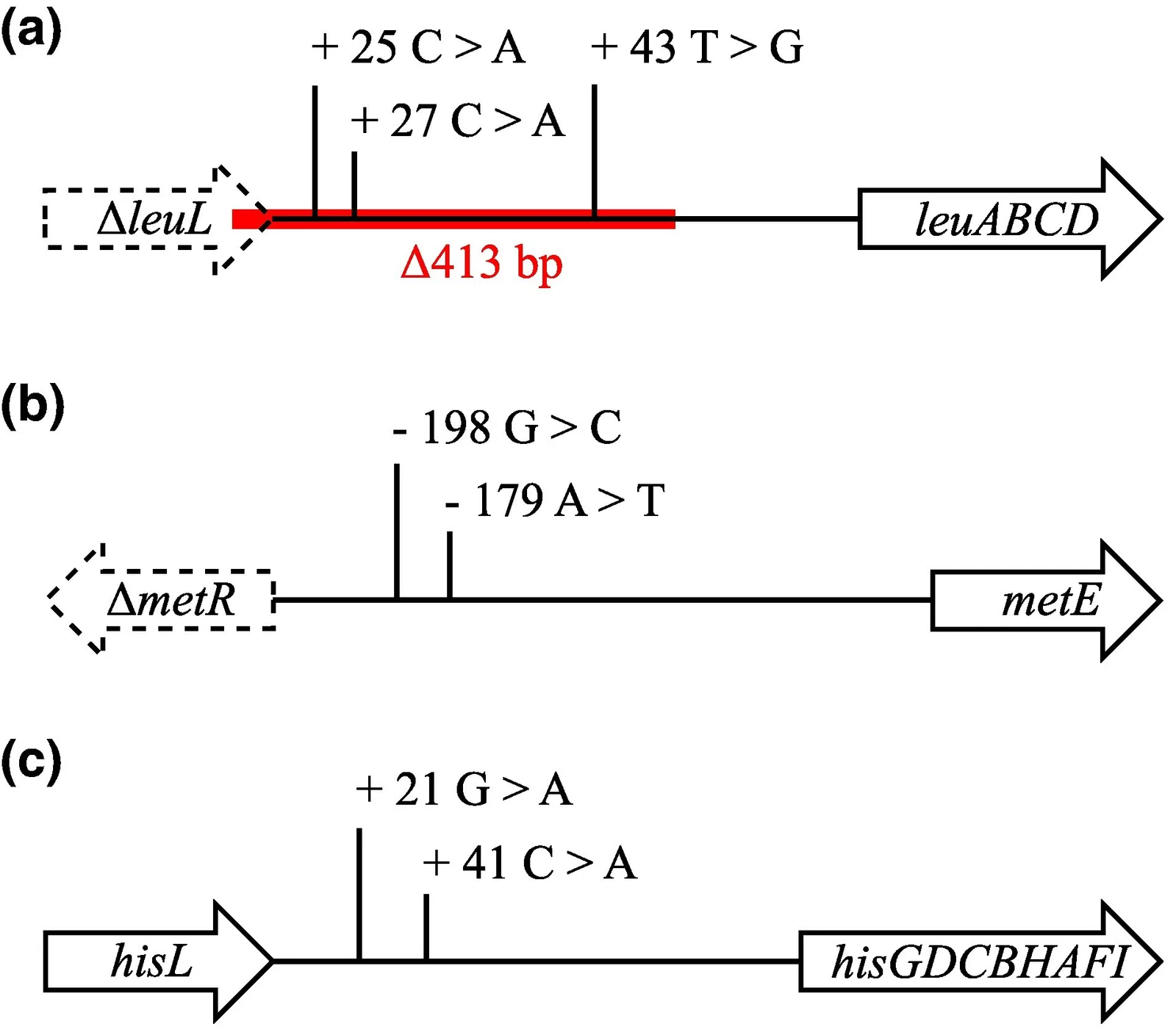
Mutations in intergenic regions causing suppression of auxotrophy. a) Map of the intergenic region upstream of the *leuABCD* operon showing the locations of four mutations causing suppression of Δ*leuL*: one deletion, shown as a thick line, and three nt substitution mutations with numbering relative to the last nt of the *leuL* stop codon. b) Map of the *metR- metE* intergenic region showing two nt substitution mutation that suppress the auxotrophy caused by Δ*metR*, relative to the first nt of the start codon of *metE*. c) Map of the intergenic region upstream of the histidine operon showing the location of two nt substitution mutations causing suppression of Δ*serB*, relative to the last nt of the *hisL* stop codon.

LeuL encodes a 28 amino acid leader peptide involved in attenuation of transcription of the *leuLABCD* operon in response to leucine abundance (Wessler and Calvo 1981). The leucine auxotrophy of the mutant suggests that there is insufficient transcription of the operon to support growth as a result of the deletion of *leuL*. Note that the deletion mutations in the Keio collection were constructed in-frame so as not to cause polarity effects (Baba et al. 2006). Accordingly, transcripts from the *leuL* promoter will be unregulated due to the absence of translational pausing and transcription will terminate in the *leuL*-*leuA* intergenic region. We selected seven independent suppressor mutants. Whole genome sequencing showed that six of them carried one of three different nucleotide substitution mutations mapping in the intergenic region downstream of *leuL* (+25, +27, and +43), all within the predicted stem-loop for rho-independent transcription termination (Fig. 3a and Table S7). The remaining suppressor carried a deletion-fusing sequence within the *leuL*::kan-cassette sequence to sequence upstream of *leuA* (Table S7). We propose that the selected suppressor mutations each act by disrupting the stem-loop structure that promotes transcription termination, allowing transcriptional readthrough and constitutive expression of the leucine operon, thus suppressing the auxotrophy (Fig. S7).

### Suppression of Δ*metR*

The MetR protein is a DNA-binding positive transcriptional regulator of several genes involved in methionine biosynthesis, including the divergently transcribed gene, *metE* (Weissbach and Brot 1991). We isolated six independent suppressors, each of which was found by whole genome sequencing to have a nucleotide substitution mutation at either of two positions in the intergenic region between *metR*—*metE* (−179, −198, relative to the start codon of *metE,* Fig. 3b). Our hypothesis is that the suppressor mutations act by enhancing RNA polymerase binding upstream of *metE*, making its expression independent of MetR protein. The methionine auxotrophy caused by the Δ*metR* mutation and its reversal by nucleotide substitution mutations in the *metR-metE* intergenic region (Table S8, and Fig. S8), is consistent with MetR being required to activate expression of *metE* (Maxon et al. 1989). This hypothesis is consistent with the observation that overexpression of *metE* on a multicopy plasmid suppresses the auxotrophy caused by deletion of *metR* (Patrick et al. 2007).

### Suppression of Δ*proA*

The second committed step in proline synthesis is the conversion of γ-L-glutamyl 5-phosphate to L-glutamate-5-semialdehyde, carried out by the product of *proA*, glutamate-5-semialdehyde dehydrogenase (Hayzer and Leisinger 1980; Fichman et al. 2015). Attempts to select suppressors of auxotrophy in the Keio Δ*proA* strain (JW0233) failed repeatedly. Our inability to select suppressors was surprising because previous studies (Kuo and Stocker 1969; Berg and Rossi 1974) reported that inactivating mutations in *argD* suppressed Δ*proA* in *Salmonella typhimurium*. The proposed mechanism was that inactivation of *argD* caused a buildup of the precursor N-acetyl-L-glutamate 5-semialdehyde (Fig. S9), which could then be deacetylated by the ArgE enzyme into L-glutamate-5-semialdehyde and fed into the proline pathway (Kuo and Stocker 1969; Berg and Rossi 1974). By sequencing the Keio strain, we discovered that in addition to Δ*proA*, it also carried a frameshift mutation in *proB* suggesting that the failure to select suppressors could be because both genes were inactivated (Table S9). To test this, we reconstructed a Δ*proA* mutation in W3100 (the Keio parental strain) and a Δ*proA*-Δ*proB* double mutant in MG1655. The genotypes were confirmed by whole genome sequencing, and selections for suppressors of auxotrophy were repeated. Suppressors were not selected in the double mutant but were easily selected in the reconstructed Δ*proA* strain. We sequenced seven independently selected suppressors of Δ*proA* (Table S9) and identified two different mechanisms of suppression. One class of Δ*proA* suppressors had probable gene inactivating mutations in *argD*, including an amino acid substitution, an IS insertion, a frameshift mutation, and a partial gene deletion. We predicted that inactivation of *argD* should be sufficient to suppress Δ*proB* and/or Δ*proA*. To test this, we constructed strains with an engineered deletion of *argD* and Δ*proB*, Δ*proA*, and Δ*proBA*. Inactivation of *argD* was sufficient to suppress the proline auxotrophy associated with deletion of *proB*, *proA*, and *proBA* (Figs. S10 and S11) in agreement with the proposed bypass mechanism of suppression (Kuo and Stocker 1969; Berg and Rossi 1974). We do not have an explanation why spontaneous *argD* mutants were not selected in the original Keio Δ*proA* strain (JW0233). In a different mechanism of suppression, two mutants acquired different single nucleotide mutations in the regulatory region upstream of *asd* (Fig. 1c) and another mutant carried a 16-fold amplification of a 51 kb chromosomal region that included the *asd* gene. The amplification was facilitated by recombination between an existing IS1 element and a second newly inserted IS1, with *asd* and other genes in between (Fig. 2c). Our hypothesis is that increased expression of *asd* is the cause of the Δ*proA* suppression. To test this, we cloned the *asd* onto pUC19 and showed that it grew on M9-glucose agar (Fig. S10). We concluded that overexpression of *asd* is sufficient to suppress the proline auxotrophy. A model to explain suppression is that overexpression of aspartate-semialdehyde dehydrogenase (product of *asd*) performs, at a low rate, the same biochemical reaction as glutamate-5-semialdehyde dehydrogenase (product of *proA*), but on a carbon chain that is one carbon shorter, Fig. S12 (Biellmann et al. 1980). This mechanism of Δ*proA* suppression was previously shown using a cloned *E. coli asd* gene (Serebrijski et al. 1995).

### Suppression of Δ*proB*

Proline synthesis in *E. coli* begins with the conversion of L-glutamate to γ-L-glutamyl 5-phosphate, a reaction carried out by ProB, glutamate 5-kinase (Hayzer and Leisinger 1980; Fichman et al. 2015). Whole genome sequencing of five independently selected suppressors of Δ*proB* revealed that each of them had acquired a mutation in *glnA* (Table S10). Each of the mutants had acquired a single amino acid substitution in GlnA, glutamine synthase, the enzyme responsible for converting L-glutamate to L-glutamine (Fig. S9). The first step in the reaction is the formation of an activated reaction intermediate, γ-L-glutamyl 5-phosphate (Eisenberg et al. 2000), which is identical to the product of the *proB*-encoded enzyme (Fig. S9). This suggests a mechanism for the suppression, namely that a premature release of a proportion of the reaction intermediate (γ-L-glutamyl 5-phosphate) from mutated glutamine synthetase could provide the substrate required to feed the proline pathway, bypassing the requirement for the ProB enzyme (Fig. S9). Similar mutations in GlnA suppressing *proB* auxotrophy have been reported by others (Medina-Carmona et al. 2023).

### Suppression of Δ*serB*

The enzyme SerB, phosphoserine phosphatase, catalyzes the last step in serine biosynthesis, the conversion of O-phospho-L-serine to L-serine (Kuznetsova et al. 2006). We selected five independent suppressors of Δ*serB* (Table S11). Two suppressors had amplified the histidine operon region by recombination between two chromosomal copies of IS5 (the same mechanism as observed for suppression of Δ*hisH*, Fig. 2d), while the other two suppressors carried different point mutations in the *hisL—hisG* regulatory region controlling transcriptional attenuation (Barnes 1978). The fifth suppressor carried a 127 nt deletion mutation in *truA*, encoding tRNA pseudouridine synthase, the enzyme responsible for catalyzing pseudouridine formation in the anticodon loop of several tRNAs including histidine tRNA (Kammen et al. 1988). What these suppression mechanisms have in common is that they are each predicted to cause upregulation of expression of the *his* operon (Winkler and Ramos-Montanez 2009). The substrates of *hisB* (L-histidinol) and *serB* (O-phospho-L-serine) share a similar structural motif suggesting a non-canonical reaction by HisB as a mechanism of suppression (Fig. S13). This suggests the hypothesis that when there is an excess of HisB, the enzyme could act on both substrates and contribute to both histidine and serine biosynthesis. In support of this, *serB-*mediated auxotrophy was previously shown to be suppressed by expression of *hisB* from a multicopy plasmid (Patrick et al. 2007). We also tested this hypothesis by deleting *hisB* in the Δ*serB* strain and showing for the double mutant, Δ*serB-*Δ*hisB* that suppressors could no longer be selected (frequency < 7.4E−11). We also constructed a Δ*serB*-Δ*truA* strain, confirming our prediction that this genotype would be prototrophic (Fig. S14).

#### Relative Growth Fitness of the Suppressor Mutants

**Fig. 4.**
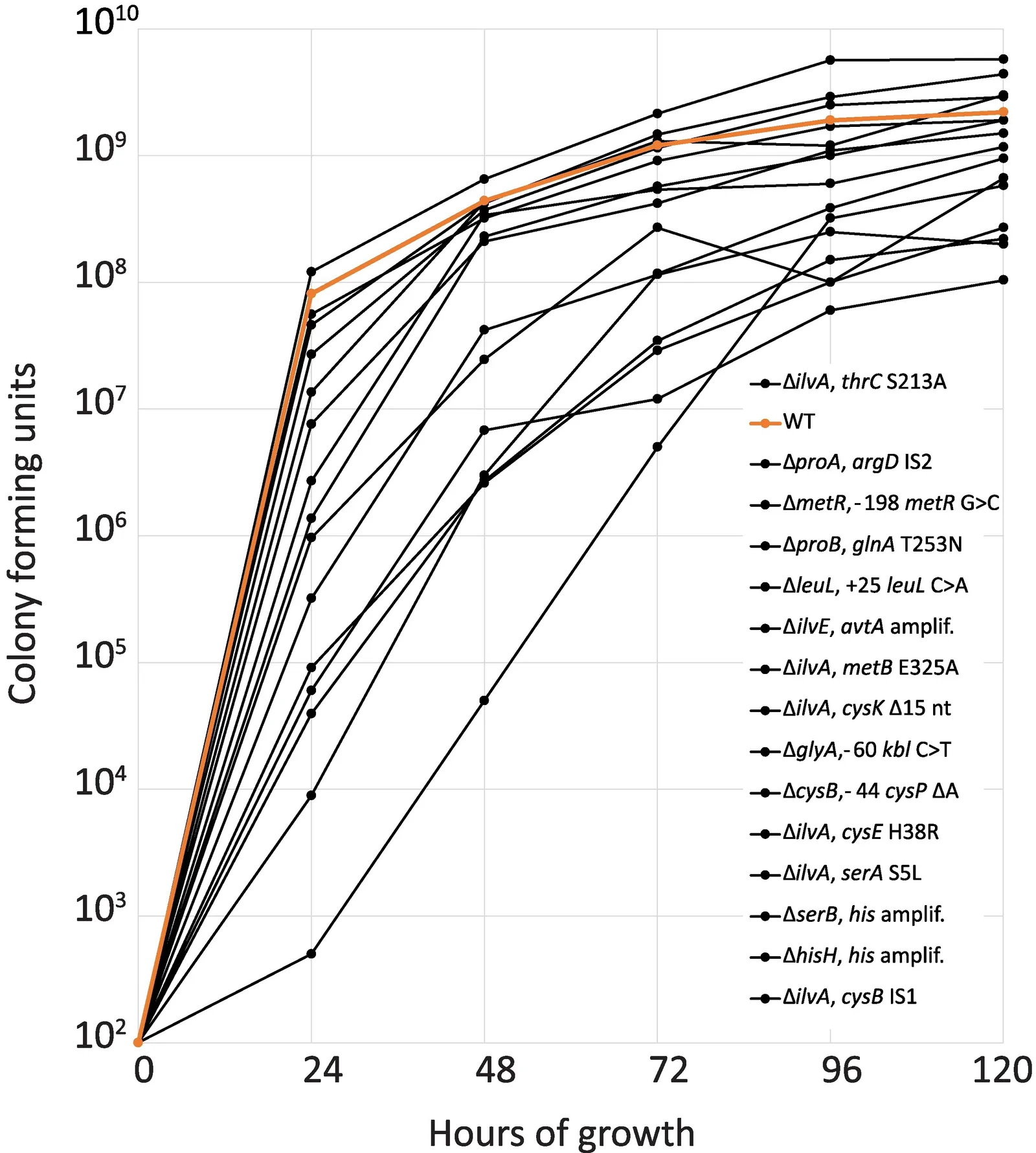
Growth rates of 15 different suppressor mutants relative to the wild-type. Growth was measured on minimal agar as the increase in CFU over time. The wild-type is shown in orange, each of the mutants in black. The genotypes of each suppressor strain are listed in order of CFU at 24 h. More complete details of the mutant strains and CFU at each time point can be found in Table S12.

While 10/59 irreversible amino acid auxotrophs could be suppressed by mutations within the genome, this does not necessarily mean that they would thrive in the face of competition. To assess this, we measured growth fitness, on minimal agar, for 15 suppressor mutants, representative of each auxotrophy and the variety of suppressor classes isolated. The results show classic growth curve kinetics tracking the increase in CFUs of each suppressor mutant over a period of 120 h. A few of the suppressors are relatively fit by this assay, growing as much, or almost as much, as the wild type in the initial 24 h growth phase (Fig. 4). However, for the great majority of the mutants, the initial 24 h increase in CFU was at least one log lower than that achieved by the wild-type strain (Fig. 4 and Table S12). Accordingly, most mutational suppressors of auxotrophy are uncompetitive in growth relative to wild-type strain. This suggests that a more realistic pathway to restoration of prototrophy would be by reacquisition of the missing gene via some mechanism of horizontal genetic transfer (HGT).

#### HGT Can Convert the Non-Suppressible Auxotrophic Mutants to Prototophy

*E. coli* is not naturally competent, but there are two established mechanisms of HGT relevant to chromosomal genes: conjugative transfer associated with chromosomally integrated conjugative elements (Hfr mechanism) and bacteriophage-mediated transfer of chromosomal DNA (generalized transduction mechanism). We tested each of these HGT mechanisms for their ability to convert each of the non-suppressible auxotrophs to prototrophy, using an *E. coli* Hfr strain, and a lysate of P1 bacteriophage grown on the prototrophic parent of the *E. coli* auxotrophs. We found that each of the irreversible auxotrophs could be made prototrophic by both conjugation and transduction (Fig. S15).

In summary, we have isolated spontaneous suppressors for 10 out of 59 irreversible amino acid auxotrophs (Table 1). The genetic mechanisms of suppression fall into several classes: tandem amplifications of regions of the chromosome associated with recombination between similarly oriented IS elements; point mutations occurring in intergenic regions affecting either promoter activity or regulator protein binding; and point mutations occurring within coding sequences to generate suppressor proteins. In terms of phenotypic mechanisms, the suppressors also fall also into some broad classes: suppression by amplification of weak intrinsic enzyme side activities; rewiring of metabolic pathways; and relaxation of enzyme specificity, as seen for suppression of Δ*proB* by *glnA* mutations.

## Discussion

Complex multi-gene biological systems carry an inherent risk of being subject to mutational inactivation. Amino acid synthesis in *E. coli*, involving 59 individually essential protein-coding genes, represents a large genetic target for inactivating mutations, including irreversible deletions. The average rate of spontaneous gene inactivation mutations in *E. coli* is in the range of 10^−7^–10^−6^ per gene per generation (Andersson et al. 2011). A naive expectation is that *E. coli*, as an asexual organism, would be subject to the effects of Muller's ratchet (Muller 1964), and accumulate deleterious mutations over time, including loss of the ability to make some of the 20 amino acids required for protein synthesis. The operation of Muller's ratchet has been demonstrated in the related bacterial species *Salmonella enterica* serovar Typhimurium, where it was found that lineages grown without the opportunity for horizontal genetic transfer (HGT) accumulated detrimental mutations including amino acid auxotrophies (Andersson and Hughes 1996). Amino acid auxotrophy is common across different bacterial phyla, usually associated with host-associated systems or habitats where amino acids are available (Ramoneda et al. 2023). Indeed, clinical isolates of Shigellae, human-host-specific close relatives of *E. coli*, are usually amino acid auxotrophs (Ahmed et al. 1988) showing that auxotrophy can evolve in *E. coli* lineages. However, natural and clinical isolates of non-*Shigella E. coli* strains are generally prototrophic, capable of growing on minimal medium with a single carbon source, perhaps reflecting that they are under selection to survive in a wide variety of habitats (Bouvet et al. 2017; Berruga-Fernández et al. 2026)

In principle, bacteria could reduce the effects of Muller's ratchet (Muller 1964) by having a large effective population size, allowing selection to operate on individuals that escape deleterious mutations, or by having a low mutation rate making it more likely that the non-mutated class is represented. Neither of these conditions is satisfied in *E. coli*: transmission to new hosts and environments frequently occurs by single individuals, conditions under which genetic drift would be high (Hannan et al. 2012; Abel et al. 2015; Garoff et al. 2020), and its mutation rate is not particularly low (Ochman and Wilson 1987; Ghalayini et al. 2018; Windels et al. 2025). One possibility to counteract the effects of Muller's ratchet is via the occurrence of compensatory mutations within the genome that ameliorate the effects of detrimental mutations. Accordingly, bacteria could, by mutational compensatory evolution, rewire their metabolic pathways in response to the loss of an essential enzyme. This idea is attractive because enzymes are not perfectly accurate and can often use alternative substrates, providing basal activities that can potentially be enhanced by amplification or point mutations (D'Ari and Casadesus 1998; Yip and Matsumura 2013; Pandya et al. 2014). This is the hypothesis that we have tested in this study, by asking to what extent *E. coli* has the ability to compensate for the phenotype of irreversible deletion mutations causing amino acid auxotrophy by some form of intracellular compensatory evolution. Finally, bacteria could also counteract the effects of Muller's ratchet by replacing the defective genes with functional copies acquired by some mechanism of HGT.

The main result from this study is that only in a minority of cases (10/59) did spontaneously occurring genetic changes within the genome allow the amino acid auxotrophic *E. coli* to regain prototrophy. Quantitatively, this result is in line with the conclusions of a study that found that overexpressing each individual *E. coli* gene, cloned on a multicopy plasmid, could reverse auxotrophy for only 11 amino acid auxotrophic mutants (Patrick et al. 2007). Between them, these two studies identified a total of only 15 amino acid auxotrophic deletion mutants that could be made prototrophic by a genetic change occurring within the genome (Table S13). In 5 instances the gene causing suppression by spontaneous mutation coincided with a gene that caused suppression of the same auxotrophy when overexpressed. The partial overlap in genetic mechanisms between two different approaches to solving the same problem raises questions about the predictability of evolution. The measured frequencies of spontaneous suppression are spread over a 500-fold range, from 7 × 10^−11^ up to 3.6 × 10^−8^ (Table 1). This dataset is probably too small to use for definitive statements on the subject, and because the observed frequencies depend on at least two independent factors: (i) the spontaneous frequency of the mutational event (amplifications are intrinsically high-frequency but only if direct repeat sequences are appropriately located, whereas for point mutations, target size, the number of different mutations causing a phenotype, plays a very important role in the frequency); (ii) differences in the relative growth fitness of the selected mutants might affect survivability and play a role in determining the observed frequency of mutants. Nevertheless, we note that the least frequent suppressor class (suppression of Δ*ilvE* by *avtA* amplification) required a highly unusual predisposing event, two independent insertions of IS1 into the chromosome. In contrast, the most frequently observed mutationally suppressed auxotrophy (Δ*proA*) occurred by three different genetic mechanisms: *argD* inactivation (gene inactivation mutations are predicted to occur at high frequency), different *asd* promoter mutations (predicted to occur at moderately high frequency because there are multiple targets for mutation), and regional amplification including *asd* (dependent on the occurrence of an IS insertion event). In addition, inactivation of *argD* was shown to have only a small growth fitness cost (Fig. 4) suggesting that the observed high frequency of Δ*proA* suppression may be predictable due to the combined effects of a high underlying frequency of suppressor mutations combined with a low fitness cost for at least some of them. Regarding the question, why the remaining 49 mutants could not be suppressed, we can speculate. One possibility, supported by single gene overexpression experiments (Patrick et al. 2007) is that for many auxotrophic mutants there is no single *E. coli* gene product that when overexpressed, can carry out the missing function. Another possibility is that rare combinations of mutations might be required to modify a gene product to permit it to have a dual function. Neither this study nor the earlier study (Patrick et al. 2007) rule out the possibility that suppression of additional auxotrophs might be possible under conditions where multi-step evolution could occur.

The mechanisms we identified that reverse auxotrophy are compatible with the expectations of the “underground metabolism” hypothesis (D'Ari and Casadesus 1998). Thus, we identified a variety of mutations (amplifications and/or regulatory mutations) that caused increased expression of enzymes able to weakly carry out the missing function; we identified mutations that caused rewiring of metabolic pathways, activating dormant or weak functions; and we identified mutations that reduced enzyme specificity, feeding the missing product into the mutant pathway to overcome the auxotrophy. However, while these mechanisms are individually interesting and show that metabolic rewiring is possible to overcome mutational defects, our major conclusion is that *E. coli* has a very limited ability to compensate by mutation for the loss of essential genes involved in amino acid synthesis.

The mutational suppression mechanisms selected were in some cases associated with large tandem amplifications which are genetically unstable unless maintained by selection. In addition, most of the mutational suppressors selected carried large growth fitness costs (Fig. 4, and Table S12). Each of these features argues against mutational suppression mechanisms as a major contributor to the evolutionary maintenance of prototrophy in *E. coli*. An implication of this conclusion is that the prototrophy of *E. coli* isolates probably depends to a large degree on the reacquisition of missing genes through some mechanisms of HGT. The importance of HGT in altering *E. coli* genomes is well established. Sequence analysis of natural isolates has shown that nucleotide changes associated with clonal divergence are 50-times more likely to have occurred by recombination (HGT) than by mutation (Guttman and Dykhuizen 1994). In this respect it is interesting that genes of the mismatch repair system, including *mutS*, are frequently lost by mutation and reacquired subsequently by HGT, resulting in genetic mosaic patterns in these genes (Denamur et al. 2000). Because *E. coli* is not naturally competent, the mechanisms responsible for HGT are most likely either bacteriophage-mediated transduction or conjugation mediated by conjugative elements such as plasmids or ICE, integrated conjugative elements (Arnold et al. 2022). Accordingly, it is possible that bacteriophage, by a generalized transduction mechanism, could transfer the missing gene from one *E. coli* strain to another and reverse the auxotrophy. The general evolutionary significance of a generalized transduction mechanism might be limited by the bacterial strain specificity associated with many bacteriophage (Battaglioli et al. 2011). An alternative possibility is that a bacterial chromosome could be mobilized and conjugatively transferred into the auxotrophic strain (by an Hfr-like mechanism) where it repairs the missing function after homologous recombination. We made experiments with bacteriophage-mediated transduction and with Hfr-mediated conjugation (Fig. S15) and showed that each of these established mechanisms of HGT can restore prototrophy and wild-type growth to all of the tested auxotrophs. We have also recently shown that many clinical isolates of *E. coli* can mobilize and transfer their chromosomal DNA into another *E. coli*, creating chromosomal hybrids in a process that is driven by ICE and/or conjugative plasmids (Berruga-Fernández et al. 2026). Conjugative hybrid formation, whether driven by ICE or conjugative plasmids, is a plausible mechanism to reverse the effects of Muller's ratchet, maintain the fitness and prototrophy of *E. coli* isolates, and simultaneously contribute to the horizontal transfer of virulence and resistance genes.

## Materials and Methods

### Bacterial Strains and Strain Construction

Strains used in this study are derived from *E. coli* MG1655 and W3110 and are described in Table S13. Mutant strains with deletions were constructed by recombineering in MG1655 or W3110 with pSIM5-Tet (CH1940 and CH12807, respectively) using the DIRex method (Nasvall 2017). The two cassettes required for the DIRex method were PCR amplified from TH10833 and TH10832 using appropriate oligonucleotides to target recombination into the specific gene to be deleted (Table S14). Constructed mutations were transferred to other backgrounds by P1 transduction (Thomason et al. 2007). pUC19 with cloned genes for overexpression was done by restriction digestion using FastDigest Pst1 and EcoR1 (Thermo Fisher Scientific Inc., United States), dephosphorylation of cleaved plasmid using FastAP Thermosensitive Alkaline Phosphatase (Thermo Fisher), and ligation done using T4 DNA Ligase (Thermo Fisher), according to the manufacturer's instructions. Plasmids were transformed into the appropriate strain, selecting on LA + ampicillin, and growth was assessed on M9-glucose agar. All constructs were initially confirmed by PCR and local sequencing across the modified region, and strains used for selection of suppressors were validated by whole genome.

### Antibiotics

Antibiotics were used at the following concentrations: tetracycline (TET) 15 mg/L, chloramphenicol (CHL) 25 mg/L, or ampicillin (AMP) 100 mg/L. Antibiotics were purchased from Sigma Aldrich.

### Bacterial Growth and Selection Conditions

The rich culture medium for bacterial growth was Luria-Bertani broth (LB: 1% tryptone, 0.5% yeast extract, 1% NaCl), and the defined minimal salt medium was M9 (6 g/L Na_2_HPO_4_, 3 g/L KH_2_PO_4_, 0.5 g/L NaCl, 1 g/L NH_4_Cl, 0.1 mM CaCl_2_, 1 mM MgSO_4_, supplemented with 0.2% glucose). The solid medium was as above with the addition of 1.5% agar. For selection of prototrophs from auxotrophic mutants, bacterial cultures were initially grown overnight in 1 mL LB, washed by centrifugation, and resuspended in 100 µL 0.9% NaCl, then spread on M9-glucose plates. Plates were incubated at 37 °C for 120 h to allow growth of suppressed mutants. Single colonies were picked and streak-purified on M9-glucose agar. Genetic alterations in the prototrophic mutants were identified by whole-genome sequencing or local sequencing as appropriate. The growth rate of suppressor mutants (selected prototrophs) was measured on M9 minimal glucose agar as follows. Each mutant was initially grown on M9 agar at 37 °C, colonies were harvested and resuspended by vortexing in 0.9% NaCl then adjusted to 0.5 McFarland using a Nephelometer. Each suspension was diluted to 10^3^ CFU/ml, then 100 μl aliquots were pipetted onto a series of 25 mm diameter nitrocellulose filters, 0.45 μm pore size (Protran BA 85, Whatman, Germany) on M9 agar plates and incubated at 37 °C for up to 120 h. Filters were removed after each 24 h, placed into Falcon tubes with 0.9% NaCl, vortexed, and spread in a dilution series onto LB agar to measure the increase in CFU for each mutant as a function of time.

### Generation of Prototrophs by Hfr-mediated Conjugation

The Hfr donor strain BW5660 (Wanner 1986) and each of the amino acid auxotrophic recipient strains were grown overnight in LB. Equal volumes of donor and recipient cultures were mixed for each conjugation experiment, and 600 μl of the mixture were spread onto an 82 mm diameter, 0.2 μm pore nitrocellulose filter (Protran BA 83, Whatman, Germany) placed on an antibiotic-free LB agar plate. This conjugation plate was then incubated overnight at 37 °C. Filters were removed using sterile tweezers and placed in 50 ml Falcon tubes containing 2 ml 0.9% NaCl solution, vortexed to resuspend all bacteria (final density 1 to 2 × 10^10^ CFU/ml), and a dilution series plated onto M9 glucose minimal medium to select prototrophs. The frequency of prototroph formation was calculated by dividing the number of colonies growing on M9 agar by the number of recipient cells plated.

### Generation of Prototrophs by Bacteriophage-Mediated Transduction

Bacteriophage P1 lysate was prepared on *E. coli* BW25113 (Baba et al. 2006), as described (Thomason et al. 2007) with the exception that the lysate was sterilized by filtration through a 0.2 μm nitrocellulose filter (Berruga-Fernández et al. 2026). Auxotrophic strains were transduced by adding 10 μl of lysate to 100 μl of fresh overnight bacterial culture grown in LB (multiplicity of infection approximately 1). The phage-bacteria mixture was incubated for 30 min at 37 °C without shaking, washed in 0.9% NaCl, then aliquots spread onto M9 plates and incubated for 2 d at 37 °C to allow growth of prototrophic transductants. As a control, aliquots of the bacterial cultures were spread on the same medium without the addition of phage.

### Polymerase Chain Reaction

Oligonucleotide primers and their specific uses are listed in Table S15. PCR reactions were done using Phusion High-Fidelity DNA Polymerase (Thermo Fisher) for strain constructions using DIRex and using Taq DNA Polymerase (Thermo Fisher) for local sequencing, each according to the manufacturer's instructions. PCR products were purified using QIAquick PCR Purification Kit (Qiagen, Germany) according to the manufacturer's instructions, and DNA concentration was determined using a NanoDrop 1000 Spectrophotometer (Thermo Fisher). Purified PCR products were prepared for local sequencing by Mix2Seq Sanger sequencing (Eurofins Genomics, Germany) according to the manufacturer's instructions. Sequence data was analyzed using version 23.0.1 of the CLC Main Workbench (CLCbio, Qiagen).

### Whole Genome Sequencing and Sequence Analysis

Genomic DNA was purified using the MasterPure Complete DNA and RNA Purification Kit (Epicentre, United States) from cells scraped from selective agar plates. Extracted DNA was eluted in Elution Buffer (Qiagen; 10 mM Tris-Cl, pH 8.5). gDNA concentration was measured using a Qubit 2.0 Fluorometer (Invitrogen, United States). DNA was diluted to a final concentration of 0.2 ng/mL in Milli-Q water (Merck KGaA, Germany). Diluted DNA samples were prepared for whole-genome sequencing according to the Nextera XT DNA Library Preparation Guide (Illumina, United States). Sequencing was performed using the 600 cycle V3 kit on a MiSeq desktop sequencer (Illumina) according to the manufacturer's instructions. Sequence data was analyzed using version 11.0 of the CLC Genomics Workbench (CLCbio, Qiagen).

## Supplementary Material

evag209_Supplementary_Data

## Data Availability

The paired-end reads and assembled genomes of the strains described in the text have been deposited in the NCBI BioProjects database, BioProject ID PRJNA1456069. All other data discussed in the paper are available in the main text and supplementary materials.
