# Supplementary material for: The Evolutionary Maintenance of Amino Acid Prototrophy in Escherichia coli
Source: Genome Biol Evol. 2026 Aug 22;18(9):evag209. doi: 10.1093/gbe/evag209 (PMC13540730; doi:10.1093/gbe/evag209)
Supplement: Supplementary file 1 [file evag209_supplementary_data.docx]

Supplementary information

**The evolutionary maintenance of amino acid prototrophy in *Escherichia coli.***

Otto Lindahl^1^, Talía Berruga-Fernández^1,2^, Jivanti Soekhoe^1^, Douglas L. Huseby^1^ and Diarmaid Hughes^1,2^

^1^Department of Medical Biochemistry and Microbiology, Biomedical Center, Uppsala University, S-751 23, Uppsala, SWEDEN

^2^Uppsala Antibiotic Center, Uppsala University, Uppsala, Sweden

Corresponding author: Diarmaid Hughes, PhD

E-Mail: diarmaid.hughes@imbim.uu.se

**This supplementary information includes:**

Figures S1 to S15

Tables S1 to S15

**Figure S1**

**Fig. S1. Growth phenotypes of Δ*thrA*, Δ*metL* and Δ*pheA* mutants.** Dilutions (10-fold) starting from 7.5 x 10^3^ CFU of wild-type (prototrophic) and isogenic single-gene-deletion mutants spotted onto M9-glucose agar (M9) and LB agar (LA) plates. **A.** Panel A shows that both Δ*thrA* and Δ*metL* mutants grow on M9, while the double mutant Δ*thrA* Δ*metL* does not grow. **B.** Comparing panels A and B shows that while Δ*pheA* does not grow when 7.5 x 10^3^ CFU are plated (panel A), it does grow when ~3 x 10^7^ CFU are plated (panel B).

**Figure S2**

**Fig. S2. Growth phenotypes of Δ*cysB* and Δ*cysB* with suppressor mutations.** Spot dilution (10-fold) starting from 7.5 x 10^3^ CFU, on M9-glucose or M9-glucose with added cysteine.

**Figure S3**

**Fig. S3. Growth phenotypes of Δ*glyA* and Δ*glyA* with suppressor mutations.** Spot dilution (10-fold) starting from 7.5 x 10^3^ CFU, on M9-glucose or M9-glucose with added glycine.

**Figure S4**

**Fig. S4.** **Growth phenotypes of Δ*hisH* and Δ*hisH* with amplification suppressor.** Spot dilution series (10-fold) starting from 7.5 x 10^3^ CFU, on M9-glucose or M9-glucose with added glycine. Cassette deletion refers to a suppressor in which a partial deletion in the Keio*-kan*-cassette that replaces *hisH* occurred and this may create a promoter sequence driving overexpression of the later genes in the operon, *hisAFI*. The strain on the last row, Δ*hisH, hisF* is a double deletion mutant.

**Figure S5**

**Fig. S5. Growth phenotypes of Δ*ilvA* and Δ*ilvA* with suppressor mutations.** Spot dilution series (10-fold) starting from 7.5 x 10^3^ CFU, on M9-glucose or M9-glucose with added isoleucine. All strains shown carry Δ*ilvA*. **A.** Suppression by mutations in *thrC* or *metB*. **B.** Suppression by mutations in *serA* or *cysE*. **C.** Suppression by mutations in *cysK* (point mutation or deletion). **D.** Suppression by mutation in *cysB* (IS1 insertion). Deletion of *tdcB* and *sdaA* does not abolish suppression (all panels).

**Figure S6.**

**Fig. S6. Growth phenotypes of Δ*ilvE* and Δ*ilvD* with suppressor mutation and cloned *avtA* gene.** Spot dilution series (10-fold) starting from 7.5 x 10^3^ CFU, on M9-glucose or M9-glucose with added isoleucine, and M9-glucose with added isoleucine and valine.

**Figure S7**

**Fig. S7. Growth phenotypes of Δ*leuL* and Δ*leuL*with suppressor mutations.** Spot dilution series (10-fold) starting from 7.5 x 10^3^ CFU, on M9-glucose or M9-glucose with added leucine.

**Figure S8**

**Fig. S8. Growth phenotypes of Δ*metR* and Δ*metR* with suppressor mutations.** Spot dilution series (10-fold) starting from 7.5 x 10^3^ CFU, on M9-glucose or M9-glucose with added methionine.

**Figure S9**

**Fig. S9. Proline biosynthetic pathway.** Overview of the proline biosynthetic pathway and its connections to the glutamine and ornithine biosynthetic pathways. Proposed bypass mechanisms involving point mutations in *glnA* and inactivating mutations in *argD* are discussed in the text.

**Figure S10**

**Fig. S10. Growth phenotypes of Δ*proA* and Δ*proA* with suppressor mutations.** Spot dilution series (10-fold) starting from 7.5 x 10^3^ CFU, on M9-glucose or M9-glucose with added proline. **A.** Growth on M9-glucose (pUC19-*asd* is unstable and lost at high frequency during growth). **B.** Growth on M9-glucose with added ampicillin to select for retention of the pUC19-*asd* plasmid.

**Figure S11**

**Fig. S11. Growth phenotypes of Δ*proB* and Δ*proB* with suppressor mutations.** Spot dilution series (10-fold) starting from 7.5 x 10^3^ CFU, on M9-glucose or M9-glucose with added proline.

**Figure S12**

**Figure S12. Proline biosynthetic pathway showing *asd* bypass.** It is proposed that overexpression of Asd reveals a minor side activity of the enzyme (aspartate-semialdehyde dehydrogenase) allowing it to carry out essential steps in both the aspartate to homoserine pathway and the glutamate to proline pathway.

**Figure S13**

**Fig. S13. Suppression of Δ*serB* by overexpression of HisB.** The hypothesis is that when there is an excess of HisB (and in the absence of SerB) that the HisB enzyme can act on both substrates and contribute to both histidine and serine biosynthesis. In support of this, *serB-*mediated auxotrophy can suppressed by overexpression of *hisB* from a multicopy plasmid (see main text).

**Figure S14**

**Fig. S14. Growth phenotypes of Δ*serB* and Δ*serB* with suppressor mutations.** Spot dilution series (10-fold) starting from 7.5 x 10^3^ CFU, on M9-glucose or M9-glucose with added serine, added serine and histidine. The Δ*serA* Δ*truA* is a constructed strain to test the hypothesis that inactivation of *truA* causes suppression. The Δ*serB* Δ*hisB* is a constructed strain to test the hypothesis that suppressors are not selected in the absence of a functional *hisB* gene.

**Figure S15.**

**Fig. S15.** Repair of auxotrophic mutants by HGT (P1 phage-mediated transduction and Hfr-mediated conjugation).

**Table S1.** Frequency of prototrophic mutants suppressing amino acid auxotrophy.

| **Gene deleted** | **Strain** | **Frequency of prototrophs** | **Independent Cultures** |
| --- | --- | --- | --- |
| *argA* | JW2786 | < 7.0E-11 | 4 |
| *argB* | JW5553 | < 5.2E-11 | 5 |
| *argC* | JW3930 | < 7.0E-11 | 4 |
| *argE* | JW3929 | < 7.0E-11 | 4 |
| *argG* | JW3140 | < 7.0E-11 | 4 |
| *argH* | JW3932 | < 7.0E-11 | 4 |
| *aroA* | JW0891 | < 7.0E-11 | 4 |
| *aroB* | JW3352 | < 7.0E-11 | 4 |
| *aroC* | JW2326 | < 7.0E-11 | 4 |
| *aroD* | JW1683 | < 4.2E-11 | 6 |
| *aroE* | JW3242 | < 7.0E-11 | 4 |
| ***cysB*** | JW1267 | **3.5E-10** | **4** |
| *cysC* | JW2720 | < 7.0E-11 | 4 |
| *cysD* | JW2722 | < 7.0E-11 | 4 |
| *cysE* | JW3582 | < 3.7E-11 | 7 |
| *cysH* | JW2732 | < 7.0E-11 | 4 |
| *cysI* | JW2733 | < 7.0E-11 | 4 |
| *cysJ* | JW2734 | < 5.2E-11 | 5 |
| *cysN* | JW2721 | < 7.0E-11 | 4 |
| *glnA* | JW3841 | < 7.0E-11 | 4 |
| ***glyA*** | JW2535 | **1.0E-08** | 4 |
| *hisA* | JW2006 | < 7.0E-11 | 4 |
| *hisB* | JW2004 | < 7.0E-11 | 4 |
| *hisC* | JW2003 | < 7.0E-11 | 4 |
| *hisD* | JW2002 | < 7.0E-11 | 4 |
| *hisF* | JW2007 | < 7.0E-11 | 4 |
| *hisG* | JW2001 | < 7.0E-11 | 4 |
| ***hisH*** | JW2005 | **3.1E-10** | 5 |
| *hisI* | JW2008 | < 7.0E-11 | 4 |
| ***ilvA*** | JW3745 | **1.1E-08** | 10 |
| *ilvC* | JW3747 | < 5.2E-11 | 5 |
| *ilvD* | JW5605 | < 7.0E-11 | 4 |
| ***ilvE*** | JW5606 | **7.0E-11** | 5 |
| *leuA* | JW0073 | < 7.0E-11 | 4 |
| *leuB* | JW5807 | < 5.2E-11 | 5 |
| *leuC* | JW0071 | < 7.0E-11 | 4 |
| *leuD* | JW0070 | < 7.0E-11 | 4 |
| ***leuL*** | JW0074 | **2.0E-08** | 5 |
| *lysA* | JW2806 | < 5.2E-11 | 5 |
| *metA* | JW3973 | < 7.0E-11 | 4 |
| *metB* | JW3910 | < 7.0E-11 | 4 |
| *metC* | JW2975 | < 7.0E-11 | 4 |
| *metE* | JW3805 | < 7.0E-11 | 4 |
| *metF* | JW3913 | < 7.0E-11 | 4 |
| ***metR*** | JW3804 | **1.2E-09** | 7 |
| ***proA*** | CH12892 | **3.6E-08** | 4 |
| ***proB*** | JW0232 | **3.3E-09** | 5 |
| *proC* | JW0377 | < 7.0E-11 | 4 |
| *serA* | JW2880 | < 7.0E-11 | 4 |
| ***serB*** | JW4351 | **8.5E-09** | 5 |
| *serC* | JW0890 | < 7.0E-11 | 4 |
| *thrB* | JW0002 | < 7.0E-11 | 4 |
| *thrC* | JW0003 | < 7.0E-11 | 4 |
| *trpA* | JW1252 | < 2.6E-11 | 10 |
| *trpB* | JW1253 | < 7.0E-11 | 4 |
| *trpC* | JW1254 | < 7.0E-11 | 4 |
| *trpD* | JW1255 | < 7.0E-11 | 4 |
| *trpE* | JW1256 | < 7.0E-11 | 4 |
| *tyrA* | JW2581 | < 5.2E-11 | 5 |

Strains with JW designation are from the Keio collection. Strain with CH designation was constructed in the wild-type Keio strain background.

**Table S2.** Δ*cysB*, spontaneously selected suppressors.

| **Strain ID** | **Suppressor mutation** | **Relative to *cysP* start codon** |
| --- | --- | --- |
| JW1267 | - |  |
| CH13605 | ΔA | nt -44 |
| CH13606 | DUP (ATAAATATGAT) | Duplication nts (-38 to -48) |
| CH13607 | DUP (ATATGAT) | Duplication nts (-38 to -44) |
| CH13608 | DUP (ATGAT) | Duplication nts (-38 to -42) |

The mutated sequences are aligned in the table.

**Table S3.** Δ*glyA*, spontaneously selected suppressors, and genotype of constructed test strain.

| **Strain ID** | **Suppressor mutation** | **Relative to *kbl* start codon** |
| --- | --- | --- |
| JW2535 | - |  |
| CH13028 | T > A | nt -58 |
| CH13029 | C > T | nt -60 |
| CH13076 | Δ52 nts | nts -46 to -97 |
| CH13078 | Insertion IS2 | nt -60 |
| **Constructed test strain** | | |
| CH13344 | Δ*glyA* / pUC19-*tdh* | |

**Table S4.** Δ*hisH*, spontaneously selected suppressors, and genotype of constructed test strain.

| **Strain ID** |  |  |
| --- | --- | --- |
| JW2005 | - |  |
| **Suppression by amplification mutation (36.6 kb)** | | |
| CH11887 | 30-fold | *insH*(IS5) *yoeG yoeH yoeA insD insC yeeP isrC flu yeeR yeeS yeeT yeeU cbtA yeeW yoeD yoeF yeeX yeeA sbmC dacD sbcB yeeD yeeE plaP yoeI yeeY yeeZ yoeB yefM hisL hisG hisD hisC hisB* Δ*hisH hisA hisF hisI cld ugd gnd wbbL insH*(IS5) |
| CH11889 | 20-fold |  |
| CH12269 | 16-fold |  |
| CH12270 | 10-fold |  |
| **Suppression by deletion mutation** | | |
| CH12271 | Δ810 nts | Partial deletion of kanamycin cassette, contained within Δ*hisH*::*kan* |
| **Constructed test strain** | | |
| CH12897 | Δ*hisH* Δ*hisF* | |

**Table S5.** Δ*ilvA*, spontaneously selected suppressors, and genotypes of constructed test strains.

| **Strain ID** | **Gene** | **Suppressor mutation** |
| --- | --- | --- |
| JW3745 | - | - |
| CH11879 | *thrC* | S213A |
| CH11881 | *metB* | E325A |
| CH11882 | *serA* | S5L |
| CH12048 | *cysE* | H38R |
| CH12898 | *cysK* | Duplication of 36 nts |
| CH12900 | *cysK* | Deletion of 15 nts (identical to CH12912) |
| CH12902 | *cysK* | T89P |
| CH12904 | *thrC* | S213A |
| CH12906 | *serA* | L142P |
| CH12910 | *cysB* | IS1 insertion (nt 938 of 975) |
| CH12912 | *cysK* | Deletion of 15 nts (identical to CH12900) |
| CH12915 | *cysK* | E265* (TAA), out of 324 residues |
| CH12916 | *cysB* | IS1 insertion (nt 938 of 975) |
| CH12918 | *cysE* | G223V |
| **Constructed test strains** | | |
| CH13251 | Δ*ilvA*, Δ*cysK* | |
| CH13638 | Δ*ilvA*, Δ*tdcB*, Δ*sdaA*, *thrC* S213A | |
| CH13639 | Δ*ilvA*, Δ*tdcB*, Δ*sdaA*, *metB* E325A | |
| CH13640 | Δ*ilvA*, Δ*tdcB*, Δ*sdaA*, *serA* S5L | |
| CH13708 | Δ*ilvA*, Δ*tdcB*, Δ*sdaA*, *cysE* H38R | |
| CH13709 | Δ*ilvA*, Δ*tdcB*, Δ*sdaA*, *cysK* Δ15 nts | |
| CH13710 | Δ*ilvA*, Δ*tdcB*, Δ*sdaA*, *cysB* IS1 | |

**Table S6.** Δ*ilvE* and Δ*ilvD*, spontaneously selected suppressors, and genotype of constructed test strains.

| **Strain ID** |  |  |
| --- | --- | --- |
| JW5606 | - |  |
| **Suppression by amplification (16 kb)** | | |
| CH13030 | 50-fold | IS1 *xylF xylG xylH xylR bax malS* ***avtA*** *ysaA yiaJ yiaK yiaL yiaM yiaN* IS1 |
| **Constructed test strain** | | |
| CH13622 | Δ*ilvE*, pUC19-*avtA* | |
| CH13343 | Δ*ilvD*, pUC19-*avtA* | |

**Table S7.** Δ*leuL*, spontaneously selected suppressors.

| **Strain ID** | **Suppressor mutation** | **Relative to *leuL* stop codon** |
| --- | --- | --- |
| JW0074 | - |  |
| CH11875 | T > G | nt + 43 |
| CH11876 | C > A | nt + 25 |
| CH11877 | C > A | nt + 25 |
| CH11878 | Δ413 nts | ^a^nt - 364 to nt + 49 |
| CH12055 | T > G | nt + 43 |
| CH12057 | C > A | nt + 25 |
| CH12059 | C > A | nt + 27 |

^a^Deletion fuses sequence within the kan-cassette (replacing *leuL* sequence in the Keio strain) to sequence in the intergenic *leuL-leuA* region.

**Table S8.** Δ*metR*, spontaneously selected suppressors.

| **Strain ID** | **Suppressor mutation** | **Relative to *metE* start codon** |
| --- | --- | --- |
| JW3804 | - |  |
| CH11869 | A > T | nt - 179 |
| CH11908 | G > C | nt - 198 |
| CH11909 | A > T | nt - 179 |
| CH12038 | A > T | nt - 179 |
| CH12040 | A > T | nt - 179 |
| CH12042 | A > T | nt - 179 |

**Table S9.** Δ*proA*, spontaneously selected suppressors, and genotype of constructed test strains.

| **Strain ID** |  |  |
| --- | --- | --- |
| JW0233 | - | *proB* -1 FS codon 121 (CGT > CG-), Δ*proA* |
| CH12892 | - | Δ*proA* |
|  | **Gene** | **Suppressor mutation** |
| CH12936 | *argD* | IS2 insertion (aa 354 of 418) |
| CH12938 | *argD* | V151G |
| CH12940 | *argD* | T248fs |
| CH12968 | *argD* | Δ152 nts (nt 336 to 487) |
| **Suppression by amplification mutation (52.3 kb)** | | |
| CH12967 | 16-fold | IS1 *pck envZ ompR greB yhgF feoA feoB feoC yhgA bioH gntX nfuA gntT malQ malP malt rtcA rtcB rtcR glpR glpG glpE glpD yzgL glgP glgA glgC glgX glgB* ***asd*** *yhgN gntU gntK gntR yhhW yhhX yhhY yhhZ yrhA* IS1 |
|  | **Suppressor mutation** | **Relative to *asd* start codon** |
| CH12969 | ins T | nt - 74 |
| CH12970 | ins G | nt - 73 |
| **Constructed test strains** | | |
| CH11568 | Δ*proA*, Δ*argD* | |
| CH11574 | Δ*proA*, pUC19-*asd* | |
| CH11563 | Δ*proB*, Δ*argD* | |
| CH11298 | Δ*proB*, Δ*proA* | |
| CH11562 | Δ*proB*, Δ*proA,* Δ*argD* | |

**Table S10.** Δ*proB*, spontaneously selected suppressors.

| **Strain ID** |  |  |
| --- | --- | --- |
| JW0232 | - |  |
|  | **Gene** | **Suppressor mutation** |
| CH11895 | *glnA* | T253N |
| CH11897 | *glnA* | D264N |
| CH12044 | *glnA* | P184T |
| CH12045 | *glnA* | S148F |
| CH12047 | *glnA* | H172L |

**Table S11.** Δ*serB*, spontaneously selected suppressors, and genotype of constructed test strains.

| **Strain ID** |  |  |
| --- | --- | --- |
| JW4351 | - |  |
|  | **Suppressor mutation** | **Relative to *hisL* stop codon** |
| CH11891 | A > C | nt + 41 |
| CH12049 | G > A | nt + 21 |
| **Suppression by amplification mutation (36.6 kb)** | | |
| CH11893 | 34-fold | *insH(*IS5*) yoeG yoeH yoeA insD insC yeeP isrC flu yeeR yeeS yeeT yeeU cbtA yeeW yoeD yoeF yeeX yeeA sbmC dacD sbcB yeeD yeeE plaP yoeI yeeY yeeZ yoeB yefM hisL hisG hisD hisC hisB* Δ*hisH hisA hisF hisI cld ugd gnd wbbL insH(*IS5*)* |
| CH12051 | 30-fold |  |
|  | **Gene** | **Suppressor mutation** |
| CH12053 | *truA* | Δ127 nts (nt 475 to 601) |
| **Constructed test strains** | | |
| CH12889 | Δ*serB*, Δ*hisB* | |
| CH13445 | Δ*serB*, Δ*truA* | |

**Table S12.** Growth of suppressor mutants on minimal agar.

|  |  | **Increase in CFU as a function of hours incubated** | | | | |
| --- | --- | --- | --- | --- | --- | --- |
| **Strain** | **Genotype** | **24** | **48** | **72** | **96** | **120** |
| CH11879 | Δ*ilvA*, *thrC* S213A | 1.2 x 10^8^ | 6.5 x 10^8^ | 2.1 x 10^9^ | 5.7 x 10^9^ | 5.8 x 10^9^ |
| CH13238 | WT (BW25113) | 8.1 x 10^7^ | 4.4 x 10^8^ | 1.2 x 10^9^ | 1.9 x 10^9^ | 2.2 x 10^9^ |
| CH12936 | Δ*proA, argD* IS2 | 5.6 x 10^7^ | 3.2 x 10^8^ | 9.1 x 10^8^ | 1.7 x 10^9^ | 1.9 x 10^9^ |
| CH11869 | Δ*metR*, -198 *metR* | 4.6 x 10^7^ | 4.2 x 10^8^ | 1.5 x 10^9^ | 2.9 x 10^9^ | 4.4 x 10^9^ |
| CH11895 | Δ*proB, glnA* T253N | 2.7 x 10^7^ | 3.7 x 10^8^ | 1.2 x 10^9^ | 2.5 x 10^9^ | 2.9 x 10^9^ |
| CH11876 | Δ*leuL*, +25 *leuL* C > A | 1.4 x 10^7^ | 4.3 x 10^8^ | 1.3 x 10^9^ | 1.2 x 10^9^ | 3.0 x 10^9^ |
| CH13030 | Δ*ilvE, avtA* amplif. | 7.6 x 10^6^ | 2.1 x 10^8^ | 4.2 x 10^8^ | 1.1 x 10^9^ | 1.5 x 10^9^ |
| CH11881 | Δ*ilvA*, *metB* E325A | 2.7 x 10^6^ | 3.4 x 10^8^ | 5.4 x 10^8^ | 6.0 x 10^8^ | 1.2 x 10^9^ |
| CH12900 | Δ*ilvA*, *cysK* Δ15 nt | 1.4 x 10^6^ | 2.3 x 10^8^ | 5.7 x 10^8^ | 1.0 x 10^9^ | 1.9 x 10^9^ |
| CH13029 | Δ*glyA*, -60 *kbl* C > T | 9.7 x 10^5^ | 2.5 x 10^7^ | 2.7 x 10^8^ | 1.0 x 10^8^ | 6.7 x 10^8^ |
| CH13605 | Δ*cysB*,-44 *cysP* ΔA | 3.2 x 10^5^ | 4.2 x 10^7^ | 1.2 x 10^8^ | 2.5 x 10^8^ | 2.0 x 10^8^ |
| CH12048 | Δ*ilvA*, *cysE* H38R | 9.1 x 10^4^ | 2.6 x 10^6^ | 2.9 x 10^7^ | 1.0 x 10^8^ | 2.7 x 10^8^ |
| CH11882 | Δ*ilvA*, *serA* S5L | 6.0 x 10^4^ | 6.8 x 10^6^ | 1.2 x 10^7^ | 6.0 x 10^7^ | 1.0 x 10^8^ |
| CH12051 | Δ*serB, his* amplif. | 4.0 x 10^4^ | 2.7 x 10^6^ | 3.5 x 10^7^ | 1.5 x 10^8^ | 2.2 x 10^8^ |
| CH11887 | Δ*hisH, his* amplif. | 8.9 x 10^3^ | 3.0 x 10^6^ | 1.2 x 10^8^ | 3.9 x 10^8^ | 9.5 x 10^8^ |
| CH12910 | Δ*ilvA*, *cysB* IS1 | 5.0 x 10^2^ | 5.0 x 10^4^ | 5.0 x 10^6^ | 3.2 x 10^8^ | 5.8 x 10^8^ |

Population size was 1.0 x 10^2^ at time zero.

**Table S13.** Comparison of deletion mutants suppressed by spontaneous mutation (this study) versus by single gene overexpression.

| **∆*gene*** | **Frequency** | **Spontaneous mutations** | **Single gene overexpression** |
| --- | --- | --- | --- |
| *cysB* | 3.5 x 10^-10^ | *cysPUWAM* operon (promoter mutations) |  |
| *glnA* | - | - | *asnB, rsd* |
| *glyA* | 1.0 x 10^-8^ | *kbl-tdh* operon (promoter mutations) | *ltaE, tdh, yneH* |
| *hisH* | 6.7 x 10^-10^ | *his* operon region amplification (10- to 30-fold) | *hisF* |
| *ilvA* | 1.1 x 10^-8^ | *metB, serA, thrC, cysB, cysE, cysK* (mutations) | *tdcB, emrD* |
| *ilvD* | - | - | *avtA* |
| *ilvE* | 7.0 x 10^-11^ | *avtA* region amplification (50-fold) | *avtA* |
| *leuL* | 2.0 x 10^-8^ | *leuL-leuA* (intergenic mutations) | - |
| *metC* | - | - | *malY, alr, fimE* |
| *metR* | 1.4 x 10^-9^ | *metR-metE* (intergenic mutations) | *metE* |
| *proA* | 3.6 x 10^-8^ | *argD* (inactivation mutations)  *asd* region amplification (16-fold)  *asd* (upstream mutations) | - |
| *proB* | 3.3 x 10^-9^ | *glnA* | - |
| *serA* | - | - | *yneH* |
| *serB* | 8.5 x 10^-9^ | *his* operon region amplification (30-fold)  *hisL-hisG* (intergenic mutations)  *truA* (frameshift mutation) | *gph, hisB, ytjC* |
| *serC* | - | - | *yneH* |

**Table S14.** Additional strains

| **Strain** | **Genotype** | **Note** |
| --- | --- | --- |
| **TH10832** | *hisA::Acatsac1* | Template strain for *amilCP-cat'* |
| **TH10833** | *hisA::Acatsac3* | Template strain for *'cat-sacB-amilCP* |
| **CH1464** | WT | MG1655 |
| **CH1940** | pSIM5-tet | MG1655 with pSIM5-tet for lambda red |
| **CH13238** | WT | W3110 |
| **CH12807** | pSIM5-tet | W3110 with pSIM5-tet for lambda red |
| **CH12890** | *thrA::del* | W3110, constructed by lambda red |
| **CH12891** | *metL::del* | W3110, constructed by lambda red |
| **CH12895** | *pheA::del* | W3110, constructed by lambda red |
| **CH13080** | *metL::del*, *thrA::del* | W3110, constructed by P1 transduction |
| **CH11927** | *metL::del, metB* -1 FS codon 381 | W3110, JW3911 |

All other strains are described in the main text.

**Table S15. Oligonucleotides used in this study**

| **Primer** | **Sequence (5’🡪3´)** | **Function** |
| --- | --- | --- |
| cat-midF | CGACGATTTCCGGCAGTTTC | Used with Acatsac3 (TH10833) as a template and one of the locus-specific primers to generate 'cat-sacB-amilCP and one of the homology arms |
| cat-midR2 | GCCGACATGGAAGCCATCAC | Used with Acatsac1 (TH10832) as a template and one of the locus-specific primers to generate amilCP-cat' and one of the homology arms |
| cysPM-amp-F | ATCAGCTTGCCTTTATCGTG | Fw amplification of cysPUWAM promoter region |
| cysPM-amp-R | GTCTGGTATTCGCGTTAACG | Rv amplification of cysPUWAM promoter region |
| tdh-pUC-F | CATGCACTGCAGTTTACAGCTAGCTCAGTCCTAGGTATTATGCTAGCTTATCGCCTGAGGATGTGAG | Fw amplification of tdh for insertion into pUC19 |
| tdh-pUC-R | CATGCAGAATTCAATACCAGCCCTTGTTCGTG | Rv amplification of tdh for insertion into pUC19 |
| hisF-del-F | AGGCCATCGCATGCTGGCAAAACGCATAATCCCATGTCTCACACAGGGCGTGGAGATCAGGTGTAGGCTGGAGCTGCTTC | Used with cat-midF with TH10833 as a template to generate ’cat-sacB-amilCP and one homology arm for DIRex deletion of hisF |
| hisF-del-R | TGTTGTTCTGTTAACATATCCTGATCTCCACGCCCTGTGTGAGACATGGGATTATGCGTTGTGTAGGCTGGAGCTGCTTC | Used with cat-midR2 with TH10832 as a template to generate amilCP-cat’ and one homology arm for DIRex deletion of hisF |
| hisF-amp-F | TGGAACAACTGGTGGAAACC | Fw amplification hisF |
| hisF-amp-R | TTAGCACTTCGCCGGATACC | Rv amplification hisF |
| tdcB-del-F | AACGAATTAATTAAGCGTCAACGAAACCGGTGATTTGAGAAGCAACCGGCAGATCGTATGGTGTAGGCTGGAGCTGCTTC | Used with cat-midF with TH10833 as a template to generate ’cat-sacB-amilCP and one homology arm for DIRex deletion of tdcB |
| tdcB-del-R | AGGTTTTATAATGCATATTACATACGATCTGCCGGTTGCTTCTCAAATCACCGGTTTCGTGTGTAGGCTGGAGCTGCTTC | Used with cat-midR2 with TH10832 as a template to generate amilCP-cat’ and one homology arm for DIRex deletion of tdcB |
| tdcB-amp-F | CGCCCGGTGGCAATAAAA | Fw amplification tdcB |
| tdcB-amp-R | CGCGGTATGGTCGAAAAA | Rv amplification tdcB |
| sdaA-del-F | GAGTATTATCGTGATTAGTCTATTCGACATGTTTAAGGTGGGTCTGGCAATCAAAGTCCAGTGTAGGCTGGAGCTGCTTC | Used with cat-midF with TH10833 as a template to generate ’cat-sacB-amilCP and one homology arm for DIRex deletion of sdaA |
| sdaA-del-R | GTAAGAAGTATTAGTCACACTGGACTTTGATTGCCAGACCCACCTTAAACATGTCGAATAGTGTAGGCTGGAGCTGCTTC | Used with cat-midR2 with TH10832 as a template to generate amilCP-cat’ and one homology arm for DIRex deletion of sdaA |
| sdaA-amp-F | TTTTGTATGGGGAATGACCG | Fw amplification sdaA |
| sdaA-amp-R | CATTCGATTACGACGATAGG | Rv amplification sdaA |
| cysK-del-F | AGGACAGGCCATGAGTAAGATTTTTGAAGATAACTCGCTGCTCTTCACTGAGAAAGAATTGTGTAGGCTGGAGCTGCTTC | Used with cat-midF with TH10833 as a template to generate ’cat-sacB-amilCP and one homology arm for DIRex deletion of cysK |
| cysK-del-R | CAAGCTGGCATTACTGTTGCAATTCTTTCTCAGTGAAGAGCAGCGAGTTATCTTCAAAAAGTGTAGGCTGGAGCTGCTTC | Used with cat-midR2 with TH10832 as a template to generate amilCP-cat’ and one homology arm for DIRex deletion of cysK |
| cysK-amp-F | TGTTTACGATGATCCCGCTG | Fw amplification cysK |
| cysK-amp-R | GCCGCTAAAGGAAGAATTGA | Rv amplification cysK |
| avtA-pUC-F | CATGCACTGCAGTTTACAGCTAGCTCAGTCCTAGGTATTATGCTAGCAGTTCGACAGATATCCCGCT | Fw amplification of avtA for insertion into pUC19 |
| avtA-pUC-R | CATGCAGAATTCCGTAGCGCATCAGGCAATTT | Rv amplification of avtA for insertion into pUC19 |
| metL-del-F | AGGGGTAAAAATGAGTGTGATTGCGCAGGCAGGGGCGAAAGATATCAACCGGCTGGCACAGTGTAGGCTGGAGCTGCTTC | Used with cat-midF with TH10833 as a template to generate ’cat-sacB-amilCP and one homology arm for DIRex deletion of metL |
| metL-del-R | ATTTCTGAAATTACAACAACTGTGCCAGCCGGTTGATATCTTTCGCCCCTGCCTGCGCAAGTGTAGGCTGGAGCTGCTTC | Used with cat-midR2 with TH10832 as a template to generate amilCP-cat’ and one homology arm for DIRex deletion of metL |
| metL-amp-F | GGAATCATTAGGGGGAGTGG | Fw amplification metL |
| metL-amp-R | ATGGTGAGGAAAAATCAGGG | Rv amplification metL |
| proA-del-F | CAAGCCTCGTATAAATTAGCGCAACTCTCCAGCCGCGAAACCCAATGGGGCTGGAAGCACGTGTAGGCTGGAGCTGCTTC | Used with cat-midF with TH10833 as a template to generate ’cat-sacB-amilCP and one homology arm for DIRex deletion of proA |
| proA-del-R | GATCCACTTGTAAGTGGTCAGTGCTTCCAGCCCCATTGGGTTTCGCGGCTGGAGAGTTGCGTGTAGGCTGGAGCTGCTTC | Used with cat-midR2 with TH10832 as a template to generate amilCP-cat’ and one homology arm for DIRex deletion of proA |
| proA-amp-F | TGGTGATGTGATGGAAGG | Fw amplification proA |
| proA-amp-R | CAGGCGCGTTATCTTTTT | Rv amplification proA |
| argD-del-F | CATCGATATCCGCATCTTCCACCACCAGCGACGGTGCAAATTTTACCGGAATAAACTCTGGTGTAGGCTGGAGCTGCTTC | Used with cat-midF with TH10833 as a template to generate ’cat-sacB-amilCP and one homology arm for DIRex deletion of argD |
| argD-del-R | CCTGCCGATTTATGCTCCGGCAGAGTTTATTCCGGTAAAATTTGCACCGTCGCTGGTGGTGTGTAGGCTGGAGCTGCTTC | Used with cat-midR2 with TH10832 as a template to generate amilCP-cat’ and one homology arm for DIRex deletion of argD |
| argD-amp-F | CTGGCACAAGAGTATTTACAG | Fw amplification argD |
| argD-amp-R | TCATTACCAGCGTGCTTTG | Rv amplification argD |
| asd-pUC-F | CATGCAGAATTCCCAATGCTCTATTTAACTCC | Fw amplification of asd for insertion into pUC19 |
| asd-pUC-R | CATGCACTGCAGAAATGATTTCATTCAT | Rv amplification of asd for insertion into pUC19 |
| proB-del-F | GCAGAGAATCATGAGTGACAGCCAGACGCTGGTGGTAAAAGTTCACCGTGATGACATGATGTGTAGGCTGGAGCTGCTTC | Used with cat-midF with TH10833 as a template to generate ’cat-sacB-amilCP and one homology arm for DIRex deletion of proB |
| proB-del-R | AGCCTGCTCCTTAACGGGTAATCATGTCATCACGGTGAACTTTTACCACCAGCGTCTGGCGTGTAGGCTGGAGCTGCTTC | Used with cat-midR2 with TH10832 as a template to generate amilCP-cat’ and one homology arm for DIRex deletion of proB |
| proB-amp-F | TTCTGCAGCCTGTACAGATGCAG | Fw amplification proB |
| proB-amp-R | CAATAACCCCCAGCGGTACGCG | Rv amplification proB |
| hisB-del-F | AGCAAGTTTGATGAGTCAGAAGTATCTTTTTATCGATCGCACCCTGCCCTCGTCGAAAGGGTGTAGGCTGGAGCTGCTTC | Used with cat-midF with TH10833 as a template to generate ’cat-sacB-amilCP and one homology arm for DIRex deletion of hisB |
| hisB-del-R | ACCACGTTCATTACAGCACTCCTTTCGACGAGGGCAGGGTGCGATCGATAAAAAGATACTGTGTAGGCTGGAGCTGCTTC | Used with cat-midR2 with TH10832 as a template to generate amilCP-cat’ and one homology arm for DIRex deletion of hisB |
| hisB-amp-F | CCGCACTGAAAGAGATCCC | Fw amplification hisB |
| hisB-amp-R | AACAGTTTATCGGCCAGCA | Rv amplification hisB |
| truA-del-F | ATTATTCTCGTTAGTCCGCCAGAAATAGCGGGCCCATTGGATAAACTGGCGGTTGTTGCTGTGTAGGCTGGAGCTGCTTC | Used with cat-midF with TH10833 as a template to generate ’cat-sacB-amilCP and one homology arm for DIRex deletion of truA |
| truA-del-R | ATCTGTACTAATGTCCGACCAGCAACAACCGCCAGTTTATCCAATGGGCCCGCTATTTCTGTGTAGGCTGGAGCTGCTTC | Used with cat-midR2 with TH10832 as a template to generate amilCP-cat’ and one homology arm for DIRex deletion of truA |
| truA-amp-F | ACAGCAATGAATCACCCG | Fw amplification truA |
| truA-amp-R | CACTGGCAGCAGAAGAAG | Rv amplification truA |
| thrA-del-F | GTGGCAAATGCAGAACGTTTTCTGCGTGTTGCCGATATTCCAATGACGTTACAGCTGCCGGTGTAGGCTGGAGCTGCTTC | Used with cat-midF with TH10833 as a template to generate ’cat-sacB-amilCP and one homology arm for DIRex deletion of thrA |
| thrA-del-R | TAGCAGATCAGCAAAGACACCGGCAGCTGTAACGTCATTGGAATATCGGCAACACGCAGAGTGTAGGCTGGAGCTGCTTC | Used with cat-midR2 with TH10832 as a template to generate amilCP-cat’ and one homology arm for DIRex deletion of thrA |
| thrA-amp-F | GGCAATATGTCTCTGTGTGG | Fw amplification thrA |
| thrA-amp-F | GCATATTCTTTTCCAGGGTCA | Rv amplification thrA |
| pheA-del-F | CGAGAGAAAATCAGCGCGCTGGATGAAAAATTATTAGCGTAATCACCCGTTCAATGAAGGGTGTAGGCTGGAGCTGCTTC | Used with cat-midF with TH10833 as a template to generate ’cat-sacB-amilCP and one homology arm for DIRex deletion of pheA |
| pheA-del-R | ACTTGGGTAACAGCCCAATACCTTCATTGAACGGGTGATTACGCTAATAATTTTTCATCCGTGTAGGCTGGAGCTGCTTC | Used with cat-midR2 with TH10832 as a template to generate amilCP-cat’ and one homology arm for DIRex deletion of pheA |
| pheA-amp-F | GAAGTTGAAGAAGAGTAGTCC | Fw amplification pheA |
| pheA-amp-R | AAGCAGGCGTTTATTGACAG | Rv amplification pheA |
